# Unveiling the Nutritional Veil of Sulforaphane: With a Major Focus on Glucose Homeostasis Modulation

**DOI:** 10.3390/nu16121877

**Published:** 2024-06-14

**Authors:** Yanan Wang, Xiaoyun He, Nan Cheng, Kunlun Huang

**Affiliations:** 1 Key Laboratory of Precision Nutrition and Food Quality, Key Laboratory of Functional Dairy, Ministry of Education, College of Food Science and Nutritional Engineering, China Agricultural University, Beijing 100083, China; wangyannan_cau@126.com (Y.W.); hexiaoyun@cau.edu.cn (X.H.); goodluckchengnan@163.com (N.C.); 2 Key Laboratory of Safety Assessment of Genetically Modified Organism (Food Safety), The Ministry of Agriculture and Rural Affairs of the P.R. China, Beijing 100083, China

**Keywords:** sulforaphane, glucose homeostasis, *in vitro*/*in vivo*, human trials, bioavailability, safety

## Abstract

Abnormal glucose homeostasis is associated with metabolic syndromes including cardiovascular diseases, hypertension, type 2 diabetes mellitus, and obesity, highlighting the significance of maintaining a balanced glucose level for optimal biological function. This highlights the importance of maintaining normal glucose levels for proper biological functioning. Sulforaphane (SFN), the primary bioactive compound in broccoli from the Cruciferae or Brassicaceae family, has been shown to enhance glucose homeostasis effectively while exhibiting low cytotoxicity. This paper assesses the impact of SFN on glucose homeostasis *in vitro*, *in vivo*, and human trials, as well as the molecular mechanisms that drive its regulatory effects. New strategies have been proposed to enhance the bioavailability and targeted delivery of SFN in order to overcome inherent instability. The manuscript also covers the safety evaluations of SFN that have been documented for its production and utilization. Hence, a deeper understanding of the favorable influence and mechanism of SFN on glucose homeostasis, coupled with the fact that SFN is abundant in the human daily diet, may ultimately offer theoretical evidence to support its potential use in the food and pharmaceutical industries.

## 1. Introduction

Blood glucose homeostasis is crucial for the maintenance of normal biological functions in the human body. Disruption of glucose homeostasis can lead to various metabolic syndromes and complications, such as cardiovascular disease [1], hypertension [2], type 2 diabetes mellitus (T2D) [3], obesity [4], nephropathy [5], nonalcoholic fatty liver disease (NAFLD) [6], diabetic foot ulcer [7], and cancer [8]. Although there are numerous strategies for preventing and managing glucose metabolic disorders, such as medications and exercise, it is important to note that some of these methods may require consistent effort and could potentially have adverse effects [9,10,11]. There is growing evidence indicating that numerous naturally occurring bioactive phytochemicals have the potential to mitigate glucose metabolic dysfunction and its associated complications, for example, berberine [12], resveratrol [13], polyphenols [14], and curcumin [15]. These natural compounds possess low toxicity and exhibit highly beneficial biological effects, making them suitable for use as dietary supplements to improve glucose homeostasis [16].

Cruciferae or Brassicaceae, which include plants like broccoli, cabbage, cauliflower, and kale, are an excellent source of bioactive ingredients, such as isothiocyanates, ascorbic acid, essential minerals, and polyphenols [17]. The potential of broccoli to prevent and combat metabolic disturbance has led to its recognition as a promising candidate for functional foods. For example, research revealed that broccoli microgreen juice supplementation significantly decreases body weight by altering gut microbiota and improving insulin sensitivity in high-fat diet (HFD)-induced C57BL/6J obese mice [18]. Dietary supplementation with 10% (*w*/*w*) broccoli florets or broccoli stalks has also been shown to reduce proinflammatory factors, improve insulin sensitivity, and increase liver fatty acid oxidation by modulating the intestinal microbiome, specifically by increasing *Akkermansia muciniphila* and decreasing *Mucispirillum schaedleri* abundance [19,20]. A systematic review offers fresh perspectives into preclinical approaches for managing conditions such as cardiovascular disease, obesity, diabetes, hypertension, and NAFLD using sulforaphane (SFN) supplements [21]. Hence, there is widespread acceptance that SFN-containing foods are beneficial for improving multiple metabolic diseases.

SFN (1-isothiocyanato-4-(methylsulfinyl)-butane), a naturally occurring isothiocyanate in cabbage and broccoli with a molecular weight of 177.29 g/mol and a slight yellow lipid appearance, has been utilized as a dietary supplement to ameliorate metabolic disturbances [22]. Studies on the mechanism have demonstrated that SFN is capable of ameliorating metabolic syndromes by activating nuclear-factor-erythroid-2-related factor 2 (Nrf2) or effectively modulating the AMP-activated protein kinase (AMPK) signaling pathway [23,24]. Increasing evidence has established a connection between the consumption of dietary SFN and the improvement of abnormal glucose metabolism [25,26,27]. In the current study, our objective is to conduct a systematic review of the modulatory impact of SFN on glucose homeostasis and the underlying mechanisms, utilizing *in vitro*, *in vivo*, and human trials.

**Figure 1:**
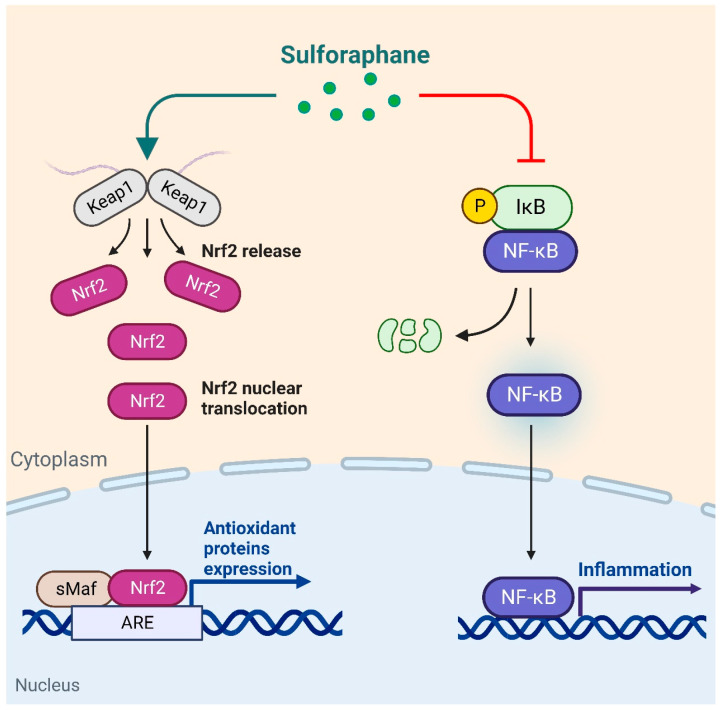
Schematic representation of signaling pathways regulated by SFN against inflammation and oxidation. SFN activates Nrf2, promotes Nrf2 translocation into the nucleus, and subsequently promotes antioxidant gene expression. In addition, SFN inhibits the expression of NF-κB, reduces the expression of inflammatory genes, and reduces inflammation in the whole organism.

It has been widely reported that SFN can modulate glucose homeostasis through the Nrf2 signaling pathway [28,29] (Figure 1). Nrf2, as a transcription factor, plays a critical role in the regulation of cellular defense mechanisms against toxic and oxidative stressors [30,31]. It achieves this by controlling the expression of genes implicated in the response to oxidative stress and the detoxification of drugs. Activation of Nrf2 leads to enhanced cellular resistance against chemical carcinogens and inflammatory challenges [32]. Furthermore, apart from its involvement in antioxidant responses, Nrf2 also contributes to a multitude of other cellular processes, including metabolism and inflammation [30,33]. Nrf2 plays an important role in many tissues, including the liver [34,35], adipose tissue [36], and heart [37]. SFN is characterized as a potent and remarkably selective Nrf2 agonist that binds to specific DNA sequences and promoting the expression of antioxidant and detoxification enzymes, protecting cells from damage caused by oxidative stress and inflammation [38]. Research demonstrated that the genetic depletion of Nrf2 in wild-type mice led to disrupted glucose metabolism, and this imbalance could not be mitigated by SFN administration in Nrf2 knockout (Nrf2^−/−^) mice. These findings indicate that SFN regulates glucose homeostasis through the Nrf2 signaling pathway [39].

## 2. Methodology

The present review proposes the concept of gathering research papers, review articles, and web-based information utilizing specific keywords, including “SFN”, “glucose homeostasis”, “therapeutic potential”, “natural sources of SFN”, “diabetes”, “obesity”, “cell model”, “animal model”, and “preclinical studies on SFN”. Research articles centered on the intervention of SFN for glucose homeostasis and the mechanisms underlying blood glucose regulation were systematically collected using diverse search engines, including PubMed, Wiley, Web of Science, Springer, Google Scholar, and Scopus. Additionally, scientific publications from libraries were utilized through a qualitative systematic review approach to gather pertinent and up-to-date information on SFN, guided by the aforementioned keywords. From the collected articles, we selected manuscripts that aligned with the aim and objective of this review. Our inclusion criteria required the reports to be published in English and related to the modulation of glucose metabolism, specifically focusing on the therapeutic potential of SFN and its derivatives, as well as the sources used for the production of SFN. The methodology employed resulted in the discovery of the pharmacological effects of SFN, highlighting its importance in regulating glucose homeostasis.

## 3. Uncovering SFN as a Nutraceutical

**Figure 2:**
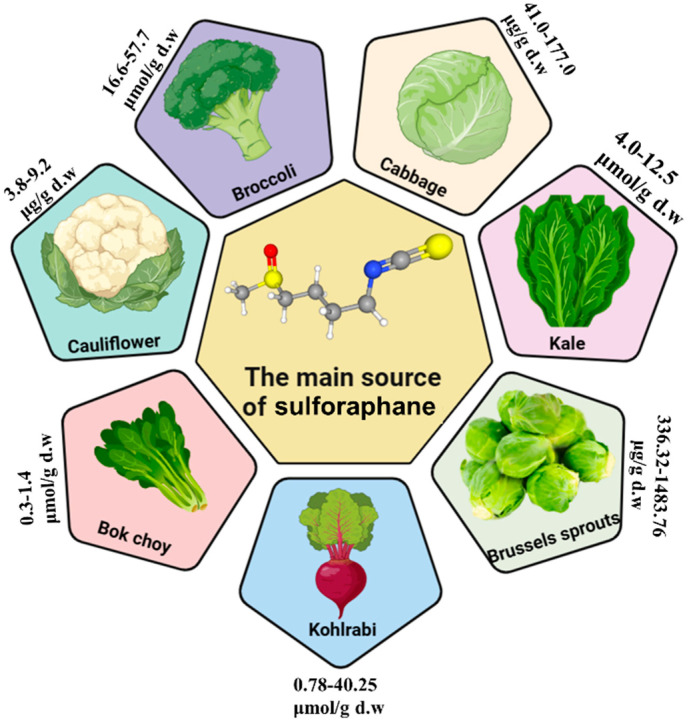
The main sources of sulforaphane include broccoli, cabbage, cauliflower, kale, bok choy, Brussels sprouts, and kohlrabi.

The Cruciferae or Brassicaceae family is renowned for its abundance of bioactive compounds, including isothiocyanates, ascorbic acid, essential minerals, and polyphenols [40]. This family includes various edible green plants like broccoli (*Brassica oleracea* var. *italica*), kale (*Brassica oleracea* var. *sabellica*), cauliflower (*Brassica oleracea* var. *botrytis*), and cabbage (*Brassica oleracea* var. *capitata*). These plants are known for their valuable compounds such as glucoraphanin [41]. Glucoraphanin, which is naturally stable and has no biological activity, undergoes a reaction with the enzyme myrosinase, a β-thioglucosidase when it is consumed by biting or chewing [42]. SFN, also known as 1-isothiocyanato-4-(methylsulfinyl) butane, is a sulfur-rich compound that is widely recognized for its antioxidant properties and ability to effectively stimulate the body’s natural detoxification enzymes [43,44,45]. It has gained significant attention and is now regarded as the most sought-after product derived from glucoraphanin due to its remarkable health benefits. As depicted in Figure 2, SFN is one of the bioactive compounds that is abundant in broccoli (16.6–57.7 μmol/g d.w) [46], cabbages (41.0–177.0 μg/g d.w) [47], kale (4.0–12.5 μmol/g d.w) [48], bok choy (0.3–1.4 μmol/g d.w) [49], cauliflower (3.8–9.2 μg/g d.w) [50], kohlrabi (0.78–40.25 μmol/g d.w) [51], and Brussels sprouts (336–1483.76 μg/g d.w) [52]. Accumulating evidence suggests that SFN has beneficial effects in ameliorating complications associated with obesity, diabetes, hypertension, and cancer, primarily through the activation of the Nrf2 signaling pathway [53,54,55,56,57]. The current pharmaceutical treatments for metabolic diseases can have side effects and may not be effective for all individuals. SFN, as a natural compound with potential therapeutic effects on glucose control and metabolic pathways, presents an opportunity for developing novel and potentially safer treatment options. Despite the availability of many pharmaceuticals to manage these conditions, there are still limitations and challenges that necessitate the exploration of alternative treatment options like SFN. Moreover, understanding the mechanisms of action of SFN in modulating glucose homeostasis and its impact on metabolic diseases is crucial for optimizing its therapeutic potential. Further research is needed to elucidate the specific pathways through which SFN exerts its effects, as well as to determine the most effective dosage, administration route, and potential interactions with other medications. Additionally, given the increasing prevalence of metabolic diseases and the growing interest in personalized and holistic approaches to healthcare, exploring the potential of SFN as a therapy for these conditions is important. Further studies can help to establish the safety, efficacy, and potential benefits of using SFN in conjunction with or as an alternative to conventional treatments, ultimately contributing to the development of more comprehensive and tailored therapeutic strategies for individuals affected by metabolic diseases.

## 4. An Overview of Blood Glucose Homeostasis and Metabolic Disease

**Figure 3:**
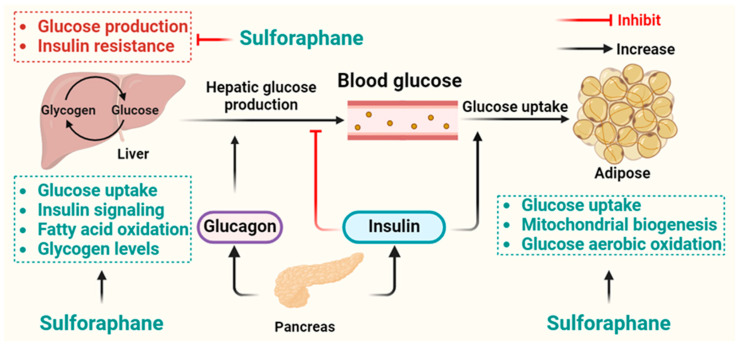
Coordination of various organs is required in the regulation of glucose homeostasis. The liver increases blood glucose levels by converting non-glucose compounds in glucose into the blood circulation through gluconeogenesis and can also achieve the conversion of glucose and glycogen according to the blood glucose level. The pancreas lowers blood glucose levels by secreting insulin that promotes glucose uptake into the adipose tissue, as well as inhibiting hepatic glucose production. In addition, in the liver, sulforaphane modulates glucose homeostasis via increasing glucose uptake, liver fatty acid oxidation, glycogen levels, insulin signaling, and inhibiting glucose production and insulin resistance. Similarly, sulforaphane also increases glucose uptake, mitochondrial biogenesis, and glucose aerobic oxidation in adipose tissues.

Blood glucose homeostasis refers to the regulation of blood glucose levels within a narrow range to ensure that cells have a constant supply of energy. Complex mechanisms of endocrine signals primarily derived from the adipose tissue, liver, pancreas, skeletal muscle, and kidney regulate glucose homeostasis [58,59,60,61,62]. This process is tightly controlled by several hormones, primarily insulin and glucagon, as well as other factors such as diet and physical activity [63,64]. When blood glucose levels rise after a meal, the pancreas releases insulin, which signals cells to take up glucose from the bloodstream and store it as glycogen in the liver and muscles. Insulin also promotes the conversion of glucose into fatty acids for storage as adipose tissue [65]. This helps to lower blood glucose levels back to normal. In order to maintain glucose homeostasis, the coordination of multiple organs is necessary, as illustrated in Figure 3.

**Figure 4:**
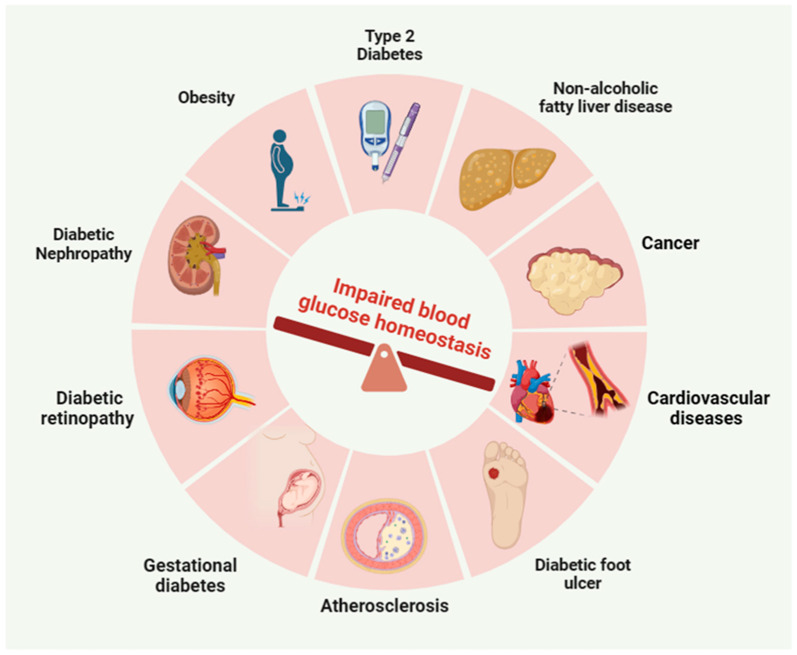
Impaired glucose homeostasis is closely related to the occurrence of metabolic syndromes including type 2 diabetes, non-alcoholic fatty liver disease, cancer, obesity, diabetic nephropathy, cardiovascular disease, diabetic retinopathy, gestational diabetes, diabetic foot ulcer, and atherosclerosis.

It is important to note that disturbances in glucose homeostasis can result in metabolic disorders like diabetes mellitus. Type 1 diabetes is characterized by the pancreas’ inability to produce insulin, leading to increased blood glucose levels. On the other hand, T2D occurs when the body develops resistance to insulin, resulting in elevated blood glucose levels. Accumulating evidence suggests that SFN has significant effects on type 2 diabetes and related complications. For example, SFN reduces hepatic glucose production and improves glucose control in patients with T2D [24]. Moreover, SFN prevents T2D-induced nephropathy via AMPK-mediated activation of lipid metabolic pathways and Nrf2 antioxidative function [56]. Both types of diabetes can have serious complications if left untreated, including cardiovascular disease, kidney damage, and nerve damage [66]. Notably, diabetic individuals are at a significantly increased risk of developing cardiovascular disease due to the presence of insulin resistance, hyperglycemia, and hyperlipidemia, which are key pathophysiological factors in the development of diabetes mellitus. SFN was found to boost Nrf2 expression and activity, indicating its potential in preventing diabetes-induced cardiomyopathy through Nrf2 modulation [67]. Similarly, Zhang et al. studied the impact of SFN on cardiac lipid accumulation in T2D and its association with diabetes-induced cardiomyopathy. They found that SFN treatment reduced cardiac remodeling and dysfunction, inhibited lipid accumulation, and improved inflammation, oxidative stress, and fibrosis in the heart [68]. Other metabolic diseases, such as metabolic syndrome and obesity, can also affect blood glucose homeostasis. Metabolic syndrome is a group of conditions characterized by high blood pressure, elevated blood glucose, excessive abdominal fat, and abnormal cholesterol levels [69]. Obesity, often associated with metabolic syndrome, can lead to insulin resistance and impair blood glucose regulation. Impaired glucose homeostasis is closely associated with the development of metabolic syndromes, as illustrated in Figure 4.

## 5. Effects of SFN on Glucose Metabolism *In Vitro*

The modulating effects of SFN on glucose homeostasis have been studied using various cell models, including liver cell lines, adipose cell lines, and other cell lines. The summarized findings of these studies are presented in Table 1.

### 5.1. Liver Cell Lines

The liver’s role in glucose metabolism is vital as it involves regulating blood glucose levels through the storage of excess glucose as glycogen and its release when glucose levels drop. Additionally, the liver is involved in gluconeogenesis, which is the production of glucose from non-carbohydrate sources like amino acids and glycerol [76]. This is important during periods of fasting or low carbohydrate intake when the body needs a steady supply of glucose for energy. Overall, the liver is a vital organ in glucose metabolism, helping to regulate blood sugar levels and ensure a constant supply of glucose for energy production in the body. HepG2 human liver cancer cells and primary mouse hepatocytes have been widely utilized as *in vitro* models for studying glucose metabolism processes and analyzing how phytochemicals can modulate insulin resistance, hyperglycemia, and diabetes [77,78].

Glucose uptake is an important process for maintaining glucose homeostasis. Regulated exocytosis of the glucose transporter GLUT4 plays a crucial role in insulin-stimulated glucose transport in adipose tissue and muscles [79]. Postprandial elevation of blood nutrients triggers insulin release, inhibiting liver gluconeogenesis and facilitating glucose absorption by muscle and adipose cells through controlled translocation of GLUT4 from intracellular reservoirs to the cell surface [80]. This process of glucose uptake is pivotal in regulating glucose homeostasis, serving as the primary mechanism for glucose utilization and storage. In recent years, there has been a growing body of research indicating that SFN may have a role in regulating glucose homeostasis through its modulation of glucose uptake in HepG2 cells. For instance, Zhang et al. showed that SFN effectively enhanced glucose uptake, enhanced insulin signaling, and induced upregulation of antioxidant genes downstream of Nrf2, leading to reduced accumulation of lipid peroxide malondialdehyde (MDA) and 4-hydroxynonenal, which was linked to the activation of the AMPK–Nrf2–Glutathione Peroxidase 4 (GPx4) axis [23]. SFN treatment improved glucose uptake and intracellular glycogen levels by modulating the insulin signaling pathway regulating phosphorylation levels of key proteins of glycogen synthesis in insulin-resistant HepG2 cells [71]. Several lines of evidence support the role of ceramides in the pathogenesis of insulin resistance [81,82]. They also showed that SFN can alleviate insulin resistance by blocking the formation of ceramide [71]. Primary mouse hepatocytes play a crucial role in glucose metabolism. These cells are responsible for regulating glucose levels in the body by taking up glucose from the bloodstream and converting it into glycogen for storage or utilizing it as an energy source. Tubbs et al. found that SFN significantly inhibits glucose production and increases glucose uptake in isolated primary mouse hepatocytes [72].

In addition, gluconeogenesis is a critical process in glucose homeostasis, allowing the liver to produce glucose when dietary sources are limited and ensuring a constant supply of glucose to meet the body’s energy demands [76]. Excessive hepatic glucose production is a significant factor in the occurrence and progression of diabetes, and its inhibition can greatly improve T2D and regulate glucose homeostasis [83,84]. Notably, SFN was found to inhibit glucose production from hepatic cells through the nuclear translocation of Nrf2 and the reduced expression of key enzymes involved in gluconeogenesis in H4IIE cells and primary mouse hepatocytes [24].

Oxidative stress plays a significant role in the development of T2D and its related complications [85]. SFN is a powerful dietary activator of Nrf2, which is the primary regulator of antioxidant cell capacity responsible for inducing cytoprotective genes [30,86]. Bernuzzi et al. reported that SFN rewires central metabolic pathways, such as glutamine and glucose metabolism, the methionine cycle, and 1C metabolism, in order to facilitate the generation of reduced glutathione and promote antioxidant responses, ultimately alleviating metabolic stress induced by excessive glucose in an Nrf2-dependent manner [70]. Taken together, SFN coordinates the regulation of hepatic glucose production, glucose uptake, and antioxidant capacity, underpinning its significant health benefits in modulating glucose homeostasis, particularly in relation to T2D.

### 5.2. Adipose Cell Lines

3T3-L1 cells are a commonly used cell line in research for the study of adipocyte differentiation and metabolism. Glucose uptake is an important process in these cells as it reflects their ability to take up and utilize glucose for energy or storage as triglycerides. Recently, Ranaweera et al. reported that SFN and broccoli leaf extract were found to stimulate glucose uptake in differentiated 3T3-L1 adipocytes [73]. Similarly, Zhang and colleagues observed an increased cellular uptake of 2-NBDG, a fluorescent deoxyglucose derivative, and significant upregulation of the expression of transporter type 4 (GLUT-4) in 3T3-L1 adipocytes treated with SFN. These findings suggest that SFN promotes glucose uptake in these cells by enhancing the expression of GLUT-4 [74]. The researchers found that SFN treatment led to a significant increase in the expressions of glucokinase and citrate synthase, which are key enzymes involved in glycolysis and the tricarboxylic acid cycle, respectively. This suggests that SFN can enhance mitochondrial biogenesis and promote the upregulation of the glucose aerobic oxidation pathway [74].

### 5.3. Other Cell Lines

Metabolic disorder, a crucial characteristic of cancer cells, enables them to rewire their metabolic pathways and adapt to the tumor microenvironment, typically characterized by limited nutrient and oxygen availability [87,88,89]. Huang conducted a recent investigation on the effect of SFN on glucose metabolism in bladder cancer cells (specifically UMUC3 cells), as well as delving into the underlying mechanism. They found that SFN exerts a strong downregulating effect on ATP production in bladder cancer cells by inhibiting both glycolysis and mitochondrial oxidative phosphorylation. Additionally, it weakens glycolytic flux through the suppression of multiple metabolic enzymes, such as hexokinase 2 and pyruvate dehydrogenase [75]. In mouse embryonic fibroblasts, SFN significantly increased the cellular glucose uptake, while this effect was abolished in Nrf2 knockout cells, demonstrating the dependency on Nrf2 and excluding any unspecific effect of the SFN [28].

## 6. Effects of SFN on Glucose Metabolism *In Vivo*

Animal models have been utilized to enhance our comprehension of glucose metabolic disorders, including diabetes, hyperinsulinemia, and obesity. These models facilitate the observation of the intricate aspects underlying the pathologies involved [90,91]. Rodents are commonly employed as animal models to explore these abnormalities due to their ready availability, short generation interval, small size, and relatively low costs [92,93]. According to animal studies, SFN could reduce glucose production and inhibit gluconeogenesis, improve glucose tolerance and insulin resistance to modulate glucose metabolism homeostasis in mouse (Table 2) and rat models (Table 3) [23,24]. The mechanisms regulating the effects of SFN on glucose homeostasis are shown in Figure 1.

### 6.1. Mouse

To investigate the potential role of SFN in modulating insulin resistance, Wu and colleagues conducted a study examining glucose homeostasis and insulin sensitivity in mice fed an HFD with or without SFN supplementation. The results revealed that mice receiving SFN supplementation exhibited significantly enhanced insulin sensitivity and improved glucose tolerance, thereby indicating its beneficial effects on obesity-related insulin resistance [94]. Dietary administration of SFN significantly improves glucose tolerance and reduces hepatic glucose production by regulating Nrf2 signaling in HFD-induced diabetic mice [24]. SFN suppresses HFD-induced body weight gain, oxidative stress, hyperlipidemia, and insulin resistance by activating the AMPK–Nrf2–GPx4 axis [23]. According to Teng, HFD-fed mice that received intraperitoneal administration of 5 mg/kg SFN for 10 weeks demonstrated ameliorated ceramide biosynthesis, reduced ceramide accumulation, and improved glucose tolerance and insulin sensitivity [71]. Tian’s study demonstrated that SFN effectively alleviated insulin resistance and enhanced glucose tolerance by reducing fasting serum glucose and insulin levels. In addition, they observed that SFN mitigated oxidative stress, adipose tissue hypertrophy, NAFLD, and inflammation. These effects were attributed to SFN’s ability to deactivate c-Jun *N*-terminal kinase, counteract the inhibitory effects on the insulin signaling pathway, and alleviate fibroblast growth factor 21 (FGF21) resistance, thus regulating glucose and lipid metabolism [95].

The metabolic changes in glucose metabolism of C57BL/6J obese-diabetic (*ob*/*ob*) mice have been extensively investigated [98]. As reported by Ranaweera et al., the blood glucose levels in *ob*/*ob* mice were significantly decreased following a six-week treatment with SFN and broccoli leaf extract [73]. Furthermore, they also discovered that the SFN content in broccoli leaf extract exhibited a promising anti-obesity effect by restoring the expression of genes associated with lipid metabolism, which were dysregulated in *ob*/*ob* mice [73]. Based on Tubbs’ findings, SFN has shown comparable effects to metformin in enhancing glucose tolerance in high-fat- and high-sucrose-diet mice and *ob*/*ob* mice. Furthermore, SFN has been observed to effectively reduce hepatic glucose production and restore disrupted mitochondria-associated ER membranes in the liver, particularly in the presence of insulin resistance [72].

In diabetes research, the STZ-induced diabetic mice model is widely employed to study the immune system’s destruction of beta cells [99,100]. Researchers can use this model to study various aspects of diabetes, including glucose metabolism, insulin resistance, beta cell function, and the complications associated with the disease [101,102]. It is also used to evaluate the efficacy and safety of potential drugs or treatments for diabetes [103,104]. According to Tian et al., in HFD and STZ-induced T2D mice, the supplementation of SFN increased serum insulin levels, HOMA-β index, and liver superoxide dismutase and glutathione peroxidase activities, while decreasing fasting blood glucose, low-density lipoprotein, liver MDA, serum total cholesterol, triglyceride, and FGF21 levels. These effects were associated with the modulation of gut microbiota [96]. 

In recent years, numerous studies have highlighted the significant efficacy of SFN in improving diabetes caused by abnormal blood glucose levels. Additionally, it has demonstrated promising preventive and ameliorative effects on diabetes-related complications [27]. A review of 34 preclinical studies was conducted to assess the potential therapeutic benefits of SFN on various complications linked to diabetes, encompassing cardiovascular problems, diabetic neuropathy, retinopathy, insulin resistance, diabetic nephropathy, skeletal muscle dysfunction, and NAFLD. For example, SFN could prevent diabetes-induced oxidative damage, inflammation, and aortic fibrosis by significantly upregulating Nrf2 expression and its downstream antioxidants [97]. Indeed, the findings from several studies also support that SFN administration can significantly reduce diabetes-related cardiovascular complications in mice [105,106]. Moreover, SFN effectively reduced diabetes-induced renal fibrosis *in vivo* by inhibiting the heightened activity of histone deacetylase 2, which was associated with increased histone acetylation and transcriptional activation of the bone morphologic protein 7 promoter [107]. Pu et al. discovered that SFN administration (1 mg/kg for 28 days) effectively alleviated cognitive decline in *db*/*db* mice by reducing levels of amyloid-beta oligomers and plaques, as well as phospho-tau in the hippocampus. This protective impact is attributed to the activation of Nrf2-regulated antioxidant defense mechanisms, leading to enhanced nuclear accumulation of Nrf2 and increased expression of antioxidant enzymes such as heme oxygenase 1 and nicotinamide adenine nucleotide phosphate quinone oxidoreductase 1, consequently resulting in decreased levels of reactive oxygen/nitrogen species in the brains of *db*/*db* mice [108]. In conclusion, the summarized literature reveals that the administration of SFN effectively mitigates oxidative stress and inflammation, thereby providing protection against the onset of diabetic-related complications. Antioxidants can help to reduce oxidative stress and inflammation in the body, which, in turn, can improve insulin sensitivity and promote better blood glucose control. By reducing inflammation, antioxidants can help to decrease insulin resistance and improve the body’s ability to regulate blood glucose levels. This can be particularly beneficial for individuals with diabetes or insulin resistance. Additionally, some antioxidants have been shown to have direct hypoglycemic effects, meaning they can help to lower blood sugar levels by promoting insulin production and uptake in cells. By targeting both inflammation and blood sugar regulation, antioxidants can play a key role in managing diabetes and supporting overall health.

### 6.2. Rat

Moreover, according to Mansour, following SFN administrations, the levels of glucose and insulin, along with the homeostatic model assessment for insulin resistance (HOMA-IR) index, exhibited significant reductions, indicating that SFN has the potential to regulate glucose metabolism through the downregulation of the phosphoinositide 3-kinase (PI3K)/protein kinase B (Akt) signaling pathway in Wistar rats [109]. De Souza et al. performed two studies to explore the beneficial impacts of SFN on STZ-induced diabetes in Wistar rats. In the first study, the rats were orally administered with daily doses of 0.1, 0.25, and 0.5 mg/kg of SFN prior to the induction of diabetes through STZ injection on the fourth day. The study lasted for 10 days to assess the effects of SFN pretreatment on the acute changes associated with the disease. The SFN-treated group showed significant reductions in hepatic glycogen concentrations, insulin sensitivity, and fasting glycemia compared to the STZ-induced diabetes Wistar rats [110]. In another study, male Wistar rats were treated with 0.5 mg/kg SFN via intraperitoneal injection for 21 days after diabetes induction. The therapeutic effects of SFN treatment on insulin sensitivity and the lipid profile of diabetic animals were demonstrated in the current study [111]. Taken together, those results indicated that SFN performs a vital role in the prevention and treatment of diabetes.

**Table 3:** The mechanisms of the modulating effect of SFN on glucose homeostasis in rat models.

Rat Models	Dosage	Method of Administration	Duration Time	Effects	Mechanisms	References
Male Wistar rats	2.5 mg/kg; three times per week	Intraperitoneal injection	15 weeks	↑ Glucose tolerance ↑ Insulin sensitivity ↓ Fasting blood glucose ↓ Hepatic glucose production	Regulation of Nrf2	[24]
Male Wistar rats	5 mg/kg; daily	Intraperitoneal injection	14 days	↑ Glucose tolerance ↓ Hepatic glucose production	Regulation of Nrf2	[24]
Male Wistar rats	0.1, 0.25, 0.5 mg/kg; daily	Intraperitoneal injection	10 days	↑ Insulin sensitivity ↑ Hepatic glycogen ↓ Fasting glycemia	/	[110]
Male Wistar rats	0.5 mg kg; daily	Intraperitoneal injection	21 days	↑ Insulin sensitivity	/	[111]
Male Wistar rats	10 mg/kg; daily	Oral gavage	4 weeks	↓ Blood glucose and insulin levels ↓ HOMA-IR index	Downregulation of PI3K/Akt signaling pathway	[109]
Goto-kakizaki rats	1 mg/kg; daily	Intraperitoneal injection	8 weeks	↑ Glucose tolerance ↓ Fasting glycemia	Regulation of Nrf2	[112]
Male Sprague–Dawley rats	0.5 mg/kg; daily	Oral gavage	44 days	↑ Glucose tolerance ↓ Fasting blood glucose and insulin levels ↓ HOMA-IR and insulin resistance	/	[113]

Abbreviation: Akt: protein kinase B; PI3K: phosphoinositide 3-kinase; Nrf2: nuclear-factor-erythroid-2-related factor 2; /: Not applicable; ↑: Increase; ↓: Inhibit.

Similarly, Axelsson and colleagues also conducted two studies to confirm the modulation effect of SFN in Wistar rats. Firstly, during the course of 15 weeks, Wistar rats were initially fed a diet consisting of 45% fat content and were simultaneously subjected to SFN treatment (2.5 mg/kg, administered intraperitoneally three times per week). The results displayed that SFN was able to increase insulin sensitivity, reduce fasting blood glucose, and improve glucose tolerance. Based on the observation that SFN can prevent diet-induced impaired glucose tolerance, the researchers proceeded to investigate its potential for treating Wistar rats that had already developed impaired glucose tolerance. Subsequently, the rats were subjected to a 60% HFD for 11 months followed by daily intraperitoneal administration of SFN (5 mg/kg) for a period of 14 days. The results obtained from this study provide evidence that SFN diminishes hepatic glucose production and enhances glucose tolerance [24].

In addition to Wistar rats, other studies have explored the impacts of SFN on glucose homeostasis in rats with other backgrounds. For example, the administration of both SFN and pyridoxamine for a period of 8 weeks demonstrated an effective reduction in circulating levels of glucose and significant improvement in glucose tolerance in Goto-kakizaki (GK) rats, which serve as an animal model for non-obese T2D [112]. In their study, Shawky et al. demonstrated that SFN has the ability to improve insulin sensitivity in fructose-fed Sprague–Dawley (SD) rats, as evidenced by reduced insulin levels and fasting blood glucose, and HOMA-IR, enhanced glucose tolerance, and alleviated insulin resistance. Furthermore, the current study provides evidence of the comparable beneficial effects of SFN to those exerted by pioglitazone, a widely used thiazolidinedione medication known for its insulin-sensitizing properties [113].

A surprising discovery was made that the long-term oral administration of SFN (1 mg/kg) in male Wistar rats fed with an HFD for four months resulted in the exacerbation of blood glucose impairment and may have influenced the expression of GLUT-3 in the cerebral cortex and hypothalamus [114]. This phenomenon deserves our deep consideration as to why it has the opposite result. It is crucial to bear in mind that the effects of natural compounds, whether beneficial or toxic, often depend on the dosage. This phenomenon can be comprehended through the concept of hormesis, which involves a biphasic response of cells or organisms to internal or external factors, such as chemical agents, oxidative stress, and dietary intake. In hormesis, the factor demonstrates beneficial or stimulatory effects at low doses, but at high doses, it exhibits inhibitory or adverse effects. Consequently, future research should explore the dose-dependent impact of SFN on the modulation of glucose metabolism within the same study.

## 7. Effects of SFN on Glucose Metabolism in Clinical Human Trials

SFN is a natural herbal drug molecule that has gained recognition for its potential role in regulating glucose homeostasis. While multiple preclinical studies have suggested its effectiveness, it is essential to conduct clinical trials to evaluate its efficacy and safety before it can be widely used for human health management. Clinical trials perform a vital role in determining the effectiveness and safety of a drug or treatment option. They provide detailed analysis and supervision, ensuring that any potential risks or side effects are properly understood and managed. Additionally, ethical principles guide the design and conduct of clinical trials, ensuring that the rights and well-being of participants are protected. By conducting well-designed clinical trials on SFN, researchers can gather scientific evidence on its effects, optimize dosage and administration, and assess its safety profile. This information is crucial for the successful development and market promotion of natural products with pharmaceutical applications. Therefore, clinical trials are crucial for the transition of natural products into pharmaceutical applications. In the following section, we will focus on SFN-related clinical research (Table 4).

For instance, a randomized controlled trial could be conducted to explore the effects of high concentrations of SFN found in broccoli sprout powder on insulin resistance in individuals with T2D. The researchers observed that after consuming 10 g/d of broccoli sprout powder for 4 weeks, there was a substantial reduction in fasting blood glucose, insulin concentration, and HOMA-IR, which indicated that broccoli sprouts may improve insulin resistance in T2D patients [116]. Meanwhile, they also found that intake of broccoli sprout powder reduced serum triglycerides, the ratio of oxidized low-density lipoprotein/low-density lipoprotein, and the atherogenic index of plasma [115]. Furthermore, a notable decline was observed in MDA, oxidative stress index, and oxidized low-density lipoprotein cholesterol, while a significant increase in serum total antioxidant capacity was also noted [117]. Taken together, these findings indicate that broccoli sprout powder exhibits beneficial effects on oxidative stress status, lipid profiles, and insulin resistance in individuals with T2D. Therefore, it can be considered an excellent candidate for managing diabetes and its related complications. In addition, Axelsson et al. conducted a double-blind, randomized, placebo-controlled study to explore the potential advantages of BSE in individuals with diabetes. They observed that in obese patients with dysregulated T2D, BSE significantly enhances fasting glucose and Hemoglobin A1c [24], whereafter a randomized controlled trial was carried out to investigate the effects of broccoli supplementation on insulin resistance in men with T2D. This study suggests that the plasma levels of insulin, HOMA-IR, and selected adipokines such as tumor necrosis factor-α (TNF-α) and interleukin 6 (IL-6) showed improvement after 12 weeks in the broccoli supplementation groups compared to their baseline levels [118]. Thorup et al. conducted a randomized, controlled, parallel-designed trial that demonstrated the significantly positive impact of consuming high amounts of root vegetables and cabbages on glucose control, insulin sensitivity, and other cardiovascular risk factors in T2D patients [119]. A recent randomized controlled cross-over study has shown that consuming vegetables first, regardless of eating speed, leads to a significantly reduced impact on postprandial blood glucose and insulin levels in young, healthy women [120].

## 8. The Advances in Increasing SFN Stabilization and Bioavailability

Despite the promising health benefits of SFN, its commercial application has been hindered by its inherent instability. Several factors such as pH, temperature, heat, light and oxygen have a profound impact on the stability of SFN, posing significant technological challenges for its stabilization. Consequently, new strategies have been proposed to enhance the bioavailability and targeted delivery of SFN in order to overcome these limitations. 

For instance, the encapsulation of nano-liposomal SFN within the hydrogel structure results in the prolonged release of SFN relative to its free form, and this effect is further enhanced as the pH levels rise [121]. A novel liposomal formulation containing both doxorubicin and SFN has been developed, demonstrating high efficiency in co-delivery to cancer cells and targeted delivery of doxorubicin to the cell nucleus [122,123]. Similarly, Mohanty et al. developed liposome–SFN combination and extensively investigated its efficacy in various *in vivo* models of acute and chronic inflammation [124]. These studies reveal the effectiveness of combining liposomes to increase the stability and water solubility of sulforaphane, which may provide an important impetus for the treatment and improvement of diseases. In addition, microencapsulation, as an important nano-particle system, is intended to protect bioactive compounds from undergoing undesirable reactions while enhancing their functionality and bioavailability [125,126]. Recently, Zambrano et al. systematically reviewed the enhancement of sulforaphane stability through microencapsulation, highlighting the superior efficacy of certain methods such as ionic gelation and complex coacervation [127]. Notably, stability investigations of microencapsulated sulforaphane across various systems are recommended, as such data will facilitate the development of sulforaphane microencapsulation approaches that broaden the industrial utility of this beneficial bioactive compound.

In summary, by encapsulating SFN in a nanocarrier, researchers have successfully achieved precise control of its release rate and biodistribution. This nanotechnology not only significantly enhanced the stability of SFN and prolonged its residence time *in vivo* but also improved its bioavailability and bioactivity, potentially mitigated the side effects, and provided new avenues for its application in drug delivery.

## 9. The Safety Evaluations of SFN

SFN has shown excellent pharmacological activity in regulating glucose homeostasis in preclinical and clinical trials. In terms of biological activity, it is essential to evaluate the toxicity potential of SFN to eliminate any potential harmful effects caused by it. Therefore, in the following part, we will focus on the safety of SFN. Socała and colleagues conducted a preliminary toxicity assessment of SFN in mice after intraperitoneal injection. They discovered that the LD_50_ value of SFN in mice was estimated to be 212.67 mg/kg, while the TD_50_ value was 191.58 mg/kg. The study revealed that administering high doses of SFN resulted in significant sedation (at doses of 150–300 mg/kg), hypothermia (at doses of 150–300 mg/kg), impaired motor coordination (at doses of 200–300 mg/kg), reduced skeletal muscle strength (at doses of 250–300 mg/kg), and mortality (at doses of 200–300 mg/kg) in mice. Furthermore, blood analysis indicated a reduction in white blood cell count (leucopenia) in mice injected with SFN at a dose of 200 mg/kg [128]. The threshold dose for side effects of SFN is approximately 10 to 20 times higher than the median dose reported for efficacy outcomes in mice, which indicated that the dose of SFN to exert its pharmacological effects was much lower than the toxic dose.

In addition, SFN is extensively utilized in cancer prevention and treatment. Studies have demonstrated its ability to mitigate the toxic effects of other anti-cancer drugs when used in combination. For instance, Kerr et al. demonstrated that the combined treatment of SFN dissolved in sterile water and delivered by oral gavage three times per week at 20 µmoles/dose, beginning 2 days after tumor cell injection, along with cisplatin effectively reduces tumor formation, invasion, and proliferation in epidermal squamous cell carcinoma [129]. Most importantly, SFN treatment demonstrated protection of H9c2 cells from doxorubicin cytotoxicity, leading to the restoration of cardiac function and a substantial decrease in doxorubicin-induced cardiomyopathy and mortality in mice [130]. In general, SFN has been shown to have minimal toxic effects when used as a pharmacological agent, but caution and close monitoring are advised for its long-term use.

## 10. Conclusions and Future Perspective

The maintenance of glucose homeostasis is a crucial physiological function in healthy individuals, involving multiple metabolic pathways. Research has shown that SFN has the ability to regulate glucose homeostasis, thereby preventing hyperglycemia and its associated complications. This study reviewed the modulating effect of SFN on glucose metabolic homeostasis, encompassing cell models, animal models, and human subjects, and evaluated the underlying mechanisms. Additionally, the safety of SFN and its various applications have also been addressed.

Despite extensive research conducted over the past few decades on the cellular and molecular mechanisms underlying impaired blood glucose, and the unequivocal demonstration of SFN’s beneficial effects on glucose homeostasis *in vitro* or *in vivo*, the development of commercially available SFN compounds for the treatment of impaired blood glucose conditions still remains a challenge. Additionally, more research is needed to explore the optimal dose and duration of SFN supplementation for different human subjects, as well as the potential interactions with other medications and supplements. Moreover, there is a need for further exploration of the molecular and cellular mechanisms that underlie the favorable impacts of SFN on metabolic disorders. This will not only deepen our understanding of the pathogenesis of metabolic diseases but will also provide a theoretical basis for the development of new drugs and therapies. In considering the use of SFN as a therapy for metabolic diseases, a potential approach could involve exploring its role in targeting key pathways and mechanisms involved in metabolic dysfunction. SFN has been shown to exhibit anti-inflammatory, antioxidant, and anti-diabetic properties, which could make it a promising candidate for addressing metabolic disorders. Utilizing SFN as a supplement or as part of dietary interventions may help improve metabolic parameters such as insulin resistance, glucose metabolism, and lipid profiles. Additionally, research on the modulation of gut microbiota by SFN highlights its potential in promoting metabolic health. Future studies could further investigate the specific mechanisms of action of SFN in metabolic diseases and explore its utility as an adjunctive therapy in combination with current treatment modalities. Overall, future research on SFN should focus on translating the promising preclinical findings into clinical practice and optimizing the therapeutic efficacy of this natural compound for the prevention and management of metabolic diseases.

To summarize, as a naturally occurring dietary bioactive compound, SFN has demonstrated its effectiveness in regulating glucose homeostasis, making it a promising candidate for dietary supplementation in the prevention of prediabetes, diabetes, and associated complications. This review serves as a valuable resource for understanding the molecular pathways related to SFN, providing a solid theoretical foundation for its potential future application in the food and pharmaceutical industries.

## Figures and Tables

**Table 1:** The mechanisms of modulating effect of SFN on glucose homeostasis in different cell models.

Cell Models	Dosage	Effects	Mechanisms	References
HepG2 cells	5, 50 nM	↑ Glucose uptake ↑ Antioxidant ↓ Insulin resistance	Activation of the AMPK–Nrf2–GPx4 axis	[23]
HepG2 cells	10 μM	↑ Glutathione biosynthesis ↑ Antioxidant	Functions in an Nrf2-dependent manner	[70]
HepG2 cells	10 µM	↑ Glucose uptake ↑ Intracellular glycogen ↓ Ceramide accumulation ↓ Insulin resistance	Modulation of the insulin signaling pathway Regulation of phosphorylation levels of key proteins of glycogen synthesis Inhibition of ceramide biosynthesis by modulating SPTLC3 expression	[71]
Primary mouse hepatocytes	3 μM	↓ Glucose production	Regulation of Nrf2	[24]
Primary mouse hepatocytes	3 μM	↑ Glucose uptake ↓ glucose production	/	[72]
3T3-L1 cells	5 µM	↑ Glucose uptake	Phosphorylation of AMPK and ACC	[73]
3T3-L1 cells	0.5, 1, 5, 10 μM	↑ Glucose uptake ↑ Mitochondrial biogenesis	Upregulation of GLUT-4 expression Upregulation of the glucose aerobic oxidation pathway	[74]
H4IIE cells	0.5 to 10 μM	↓ Gluconeogenesis	Regulation of Nrf2	[24]
Mouse embryonic fibroblasts	5 µM	↑ Glucose uptake	Functions in an Nrf2-dependent manner	[28]
HuH7 hepatocarcinoma cells	3 μM	↑ Insulin sensitivity ↓ Insulin resistance	/	[72]
UMUC3 cells	20 μM	↓ ATP production ↓ Extracellular acidification rate ↓ Bioenergetic profile oxygen consumption rate	Downregulation of both glycolysis and mitochondrial oxidative phosphorylation	[75]

Abbreviation: ACC: acetyl carboxylase; AMPK: adenosine 5′-monophosphate (AMP)-activated protein kinase; GLUT-4: glucose transporter 4; GPX4: glutathione peroxidase 4; SPTLC3: serine palmitoyltransferase, long-chain base subunit 3; Nrf2: nuclear-factor-erythroid-2-related factor 2; /: Not applicable; ↑: Increase; ↓: Inhibit.

**Table 2:** The mechanisms of the modulating effect of SFN on glucose homeostasis in mouse models.

Mouse Models	Dosage	Method of Administration	Duration Time	Effects	Mechanisms	References
C57BL/6J mice	10 mg/kg; daily	Intraperitoneal injection	4 weeks	↑ Glucose tolerance ↓ Gluconeogenesis	By regulating Nrf2	[24]
C57BL/6J mice	0.56 g/kg	Dietary supplementation	6 weeks	↑ Glucose tolerance ↓ Insulin resistance	By inducing hepatic FGF21 signaling and inhibiting p38MAPK	[94]
C57BL/6J mice	0.5, 5 mg/kg; three times per week	Intraperitoneal injection	10 weeks	↑ Insulin sensitivity ↑ Glucose tolerance ↓ Body weight gain ↓ Hepatic levels of TG, TC, ALT and AST ↓ Glycogen levels ↓ Ceramide accumulation	By regulating the IRS-1/Akt signaling pathway By downregulating SPTLC3 expression	[71]
C57BL/6J mice	10 mg/kg; daily	Oral gavage	8 weeks	↑ Glucose tolerance ↓ Insulin resistance ↓ Fasting serum glucose and insulin levels	By deactivating JNK and blocking the inhibitory effect of the insulin signaling pathway	[95]
C57BL/6J mice	10 mg/kg; daily	Oral gavage	6 weeks	↑ Glucose tolerance ↓ Hepatic glucose production	By improving disrupted ER-mitochondria interactions	[72]
C57BL/6J mice	2, 10 mg/kg; daily	Dietary supplementation	8 weeks	↑ Insulin levels, HOMA-β index, and liver SOD and GSH activities ↓ Fasting blood glucose, liver MDA, serum TC, TG, LDL-C, and FGF21 levels	By modulating gut microbiota	[96]
*Ob*/*ob* mice	0.5 mg/kg; daily	Drinking water	6 weeks	↓ TG content, LDL, cholesterol, TC, and glucose	By activating the AMPK pathway	[73]
*Ob*/*ob* mice	10 mg/kg; daily	Oral gavage	4 weeks	↑ Glucose tolerance ↓ ER stress ↓ Glucose production	By improving disrupted ER-mitochondria interactions	[72]
ICR mice	0.5 mg/kg; five times per week	Subcutaneously injection	8 weeks	↓ Body weight gain ↓ Hyperlipidemia ↓ Oxidative stress ↓ Insulin resistance	By activating the AMPK-Nrf2-GPx4 axis	[23]
FVB mice	0.5 mg/kg; five times per week	Subcutaneous injection	3 months	↓ Aortic fibrosis ↓ Inflammation ↓ Oxidative damage	By up-regulating Nrf2 and its down-stream antioxidants	[97]

Abbreviation: Akt: protein kinase B; ALT: alanine aminotransferase; AMPK: adenosine 5′-monophosphate (AMP)-activated protein kinase; AST: aspartate aminotransferase; ER: endoplasmic reticulum; FGF21: fibroblast growth factor 21; GPX4: glutathione peroxidase 4; GSH: L-Glutathione; IRS-1: insulin receptor-1; JNK: c-Jun *N*-terminal kinase; LDL: low-density lipoprotein; p38MAPK: p38 mitogen activated protein kinases; SOD: superoxide dismutase; SPTLC3: serine palmitoyltransferase, long-chain base subunit 3; MDA: malondialdehyde; Nrf2: nuclear-factor-erythroid-2-related factor 2; TC: total cholesterol; TG: triglyceride; ↑: Increase; ↓: Inhibit.

**Table 4:** The mechanisms of the modulating effect of SFN on glucose homeostasis in human subjects.

Method	Supplements	Subjects	Dosage	Duration Time	Effect	Ethical Approval No.	References
Randomized double-blind placebo-controlled clinical trial	Broccoli sprout powder	Patients with type 2 diabetes; Average age 18~60; *n* = 63	5, 10 g/d	4 weeks	↓ Fasting blood glucose and insulin levels ↓ Oxidative stress	IRCT138901181640N2.	[115,116,117]
Randomized double-blind placebo-controlled study	Broccoli sprout powder	Patients with type 2 diabetes; Average age 35~75; *n* = 97	150 mmol SFN per dose	12 weeks	↓ Fasting blood glucose ↓ Hemoglobin A1c	NCT02801448	[24]
Randomized controlled trial	Broccoli sprout powder	Patients with type 2 diabetes; Average age 40~60; Male; *n* = 44	225 µmol SFN per 10 g/d of broccoli sprout powder	12 weeks	↓ Plasma levels of HOMA-IR, insulin ↓ TNF-α and IL-6	IR-IAU1397–3	[118]
Randomized, controlled, parallel-designed trial	Brassica and root vegetables	Patients with type 2 diabetes; Average age 30~70; both sexes; *n* = 82	500 g/d	12 weeks	↑ Insulin sensitivity ↑ Glycemic control ↓ Body fat mass ↓ Blood pressure	NCT01397942	[119]
Randomized controlled cross-over study	Containing tomato, broccoli, fried fish, and boiled white rice	Healthy subjects; Average age 21.3; women; *n* = 18	/	3 weeks	↑ Postprandial blood glucose and insulin	UMIN000050266	[120]

Abbreviation: /: Not applicable; ↑: Increase; ↓: Inhibit.
